# Reliable cryo-EM resolution estimation with modified Fourier shell correlation

**DOI:** 10.1107/S2052252520011574

**Published:** 2020-09-15

**Authors:** Pawel A. Penczek

**Affiliations:** a Department of Biochemistry and Molecular Biology, The University of Texas – Houston Medical Center, 6431 Fannin Street, Houston, TX 77030, USA

**Keywords:** cryo-EM, three-dimensional structure determination, resolution, modified Fourier shell correlation, signal-to-noise ratio

## Abstract

A modified Fourier shell correlation (mFSC) methodology is introduced that is aimed at addressing two fundamental problems that mar the use of the FSC: the strong influence of mask-induced artifacts on resolution estimation and the lack of assessment of FSC uncertainties stemming from the inability to determine the associated number of degrees of freedom. It is shown that by simply changing the order of the steps in which the FSC is computed, the correlations induced by masking of the input data can be eliminated. In addition, to further reduce artifacts, a smooth Gaussian window function is used to outline the regions of reciprocal space within which the mFSC is computed. Next, it is shown that the number of degrees of freedom (ndf) of the system is approximated well by combining the ndf associated with the Gaussian window in reciprocal space with further reduction of the ndf owing to the use of the mask in real space. It is demonstrated through the application of the mFSC to both single-particle and helical structures that the mFSC yields reliable, mask-induced artifact-free results as a result of the introduced modifications. Since the adverse effect of the mask is eliminated, it also becomes possible to compute robust local resolutions both per voxel of a 3D map as well as, in a newly developed approach, per functional subunit, segment or even larger secondary element of the studied complex.

## Abstract 2

A modified Fourier shell correlation methodology is described that permits a robust, objective and reproducible assessment of resolution in single-particle cryo-EM, including helical assemblies. The method eliminates the adverse influence of masking the results. The inclusion of statistical error analysis mitigates common problems with resolution overestimation. It is shown that the method is also applicable to local resolution estimation and to resolution assessment of secondary elements such as helices and β-sheets.

## 1. Introduction   

In single-particle reconstructions, it is essential to have a robust measure of the reliability of the resulting 3D structure derived from 2D cryo-EM projection images. Cryo-EM structure determination is a complicated multi-step process that includes selection and alignment of the data (Penczek, 2008 ▸; Cheng *et al.*, 2015 ▸). A suitable reliability measure was introduced into the field early on as a 2D Fourier ring correlation (Saxton & Baumeister, 1982 ▸) and was later extended to 3D as a Fourier shell correlation (FSC; Harauz & van Heel, 1986 ▸). The principle is simple: the available 2D data are randomly split into nonoverlapping half-sets, the structure-determination process is carried out independently and the two results are correlated in reciprocal space shell by shell to yield a 1D curve of FSC coefficients as a function of the modulus of spatial frequency. High values indicate consistent Fourier components within a given shell, while those close to zero mean that there is no reliable signal. A simple proportionality relationship between the FSC and the spectral signal-to-noise ratio (SSNR) facilitates interpretation and decision making: one would want to retain Fourier components with high SSNR in the final, filtered structure, down-weight those with low SSNR and entirely eliminate those with an SSNR close to zero, which is based on the sensible rationale that one would not want to add noise to the result. Thus, it can be argued that the shape of the entire FSC/SSNR curve is an indicator of the structure quality. However, this would make it difficult to compare different results and it is preferable to have a single numerical indicator. Such a number is determined from the FSC curve as the spatial frequency at which the curve drops below a predefined threshold and is called the resolution (and is typically given in units of inverted spatial frequency, thus length). The procedure is appealing in its simplicity as the complex notion of the structure quality is reduced and expressed by a single number. However, it is apparent that two different structures can have the same resolution but quite different quality, as the latter is given by all FSC coefficients, not just one point that might be shared between two different results (Penczek, 2010 ▸).

Currently, the FSC is the only resolution measure that is commonly used in cryo-EM. While in the past other measures have been introduced, for example the 2D SSNR (van Heel & Hollenberg, 1980 ▸), *Q*-factor (Unser *et al.*, 1987 ▸) and differential phase residual (Frank *et al.*, 1981 ▸; this latter was for a while commonly used in 2D analysis), the eventual exclusive adoption of the FSC is understandable given its robustness, its ease of calculation and, most of all, its intuitively clear meaning. It is therefore somewhat surprising that despite its history of over three decades, it is still a subject of research and vigorous discussion both in publications and particularly on various social media. The controversies include both the computational methodology as well as the interpretation of the results. A closer look at the definition and in particular at calculation methods reveals that these discussions are indeed justified as, despite its simplicity, the usage of the FSC is marred by the subjective decisions that one has to make even for the simplest applications. In addition, the situation has been further complicated by the introduction of various ‘local’ resolution measures (Kucukelbir *et al.*, 2014 ▸), only some of which are based on the FSC (Cardone *et al.*, 2013 ▸) (see Vilas *et al.*, 2020 ▸).

FSC controversies can be broadly divided into two categories: (i) meaning and (ii) calculation methods. It is common to interpret the FSC outcome as an indicator of the ‘resolution’ of the structure, even though the ‘meaning’ of the FSC is not resolution as understood in optics or more broadly as a characteristic of imaging or sampling systems. The FSC resolution is simply the spatial frequency at which its value is above some preselected threshold; thus, it is obviously subjective. In addition, depending on this threshold and the way that the final map is filtered, this may or may not correspond to the highest spatial frequency Fourier components included in the map. Thus, it is not necessarily the spatial frequency limit of the map. It also follows that such a resolution cannot be interpreted as a smallest distance between details that can be resolved as separate, as is the case for the resolution as understood in optics. To an extent, this problem is caused by the lack of agreement about an acceptable threshold. Three thresholds are so far commonly used: (i) the 0.5 threshold, which is based on the corresponding level of the SSNR being equal to one and the fact that a value of 0.5 is a common characteristic of analytical low-pass filters (Malhotra *et al.*, 1998 ▸), (ii) the 3σ threshold (van Heel, 1987 ▸), which essentially corresponds to FSC = 0, or more precisely to the FSC value which under the normality assumption is three standard deviations of its variability larger than zero, and thus the signal component as expressed by the SSNR is significantly larger than zero, and (iii) the more recent 0.143 threshold, which is not based on the SSNR level but on an even harder to justify expected level of phase error in Fourier components (Rosenthal & Henderson, 2003 ▸). The first and third criteria are often referred to as ‘constant threshold’ criteria and are criticized for neglecting statistical variability (or simply error) of the FSC estimates and thus presumably leading to an overestimation of the resolution. The 3σ criterion is argued to be superior owing to the inclusion of the statistical uncertainty of the estimate. As a result, the effective cutoff threshold (the FSC value that is determined to be significantly larger than zero) varies depending on the number of Fourier coefficients included in the calculation. It can thus be considered to be ‘adaptive’, even though the test itself is based on a constant threshold, which is simply the multiplicity of the standard deviations used and which is arbitrarily set to three. Although this approach is in principle the most sensible, in practice it is compromised by as yet insurmountable difficulties. It is intuitively clear that the numbers of degrees of freedom has to be much smaller than the number of Fourier coefficients used for the calculation of the FSC, as (i) various image-processing operations use interpolation and thus introduce correlations in the data, (ii) masking of the noise surrounding the map also introduces correlations, (iii) the statistical distribution of the FSC is normal only for an expected value of zero, while for values approaching ±1 it is increasingly asymmetric, and (iv) in practice it is impossible to achieve full statistical independence of half-maps. Interestingly, there are no convincing methods that would address these issues or even quantify their effects.

The calculation of the FSC is straightforward. (i) A 3D mask is constructed such that it follows the shape of the structure as closely as possible, while at the same time the mask values are attenuated at the edges to minimize the introduction of data correlations. Also, the overall shape is made to be as ‘simple’ as possible, as highly variable shapes increase the severity of artifacts. While this step is in principle optional, in practice it is all but necessary as otherwise the computed FSC values are unrealistically low owing to the presence of background noise. Sometimes, as a compromise, spherical masks are used. (ii) Both maps are multiplied by the same mask. (iii) 3D FTs of both maps are computed. (iv) A Fourier shell width is selected and the FSC is computed, yielding its values as a function of a modulus of spatial frequency. (v) In some implementations the result is supported by an error analysis, which normally requires knowledge of the number of degrees of freedom within each shell. The main issue with this straightforward strategy is the application of the mask. Firstly, it introduces the same ‘shape’ into both maps; secondly, it introduces correlations between Fourier components whose range and strength depend on the shape and attenuation of the edges of the mask. Even more challenging is the determination of the number of degrees of freedom (ndf) to assess the significance for computed FSC coefficients. The ndf is sometimes referred to as the ‘effective sample size’, which is rather misleading. So far, no methods have been put forward to quantify these effects. Some measure of the impact can be obtained by repeating the FSC calculation using a progression of smooth-edge spherical masks with increasing radius. As the outcome of the FSC calculation using the existing protocol depends so dramatically on the mask used, it puts into question its value as an objective measure of the quality of a structure determined by single-particle cryo-EM analysis. Indeed, it is well known that unless both half-maps are given and, more importantly, the mask is supplied, it is impossible to obtain the same FSC curve as reported with the map and, in many cases, it is also difficult to obtain the same resolution value associated with the map. Since the construction of a mask given a 3D density map is not exactly an exact science, the FSC result is essentially irreproducible unless a detailed description of the mask-design protocol is provided, Worse, as there is no objective way to determine which mask is ‘better’ or more suitable, in practice it means that a consensus resolution cannot be agreed upon, as different researchers have different favorite protocols for mask design.

Of increasing interest, therefore, is the determination of the local resolution of 3D maps. Since cryo-EM analyses yield projection images of macromolecular complexes in close to the native state, it is to be expected that many reconstructed maps will show evidence of structural flexibility. Similarly, substoichiometric ligand binding adversely affects the alignment of data and therefore the local resolution. The application of an FSC for local resolution estimation of a map is however marred with difficulties. Typically, the FSC would be computed within boxes centered on all voxels within a region of interest (Cardone *et al.*, 2013 ▸). This direct approach suffers from three limitations. (i) The small size of the real-space box severely limits the spectral resolvability of the FSC estimate. (ii) The spatial resolvability can be improved by padding small boxes with zeros to a larger size, but this suffers from artifacts induced by the sharp edges of the box. The problem can be remedied by real-space window functions that taper the signal at the edges, but this compromises the spatial resolvability. (iii) Using a small number of voxels within the box dramatically increases the error of the FSC estimate. The estimate can be improved by increasing the box size, but this in turn decreases the spatial localization of the resolution estimate. Cardone and coworkers used window sizes of about 1/5 to 1/10 of the volume size, which might be sufficient to estimate the resolution of a region or a subunit of a complex, but not of small elements. An interesting and efficient approach has recently been proposed by Kucukelbir *et al.* (2014 ▸). However, the outcome is not a resolution as understood in the EM field. In particular, the result is computed in wavelet bases, not in Fourier terms, as is performed in the FSC approach. Finally, the results obtained have to be ‘calibrated’ by the variance of the noise present in the data, but the sample of this noise is obtained from the region surrounding the structure which, as is shown here, is normally higher than that within the structure and the proportionality factor is unknown. Therefore, we propose a local resolution-estimation method that is rooted in, and thus directly related to, the FSC/SSNR methodology.

In the following, we will demonstrate that the adverse impact of the mask on FSC estimation can be all but eliminated by a simple change in the order of calculations. Next, we will show that the ndf can be approximated well by accounting for the influence of the window function in reciprocal space and the number of nonzero coefficients of the real-space mask used. We will also demonstrate that in all but extreme cases FSC significance tests only mildly depend on the precise value of the ndf and thus the proposed method is acceptable for our purpose. We will then extend our objective mFSC method to an estimation of local resolution that is also mask-independent and yields reliable and detailed results.

## 2. Methods   

### 2.1. Notation   

The lower-case letters *k*, *c*, *s* denote integers or real-space numbers (scalars); they also denote real-space (discrete) functions *u*, *v*, *m*, *f*, *r*.

The bold letters **s**, **x** denote real vectors.

|.| denotes an absolute value of a scalar.

||.|| denotes the Euclidean norm of a vector, *i.e.* the square root of the sum of the squares of the elements of the argument.

Upper-case letters denote complex functions *U*, *V*, *M*.

Fourier transforms (FT) are denoted by a carat, 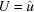, and the inverse FT is 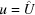.

An asterisk (*) denotes the complex conjugate of a complex variable or function.

*uv* is the inner product of two real vectors or vector representations of two volumes (*i.e.* the vector containing all elements of a discrete volume in an arbitrary order), that is the sum of pairwise products of respective elements, and the result is a scalar.

UV* is the inner product of two complex vectors or vector representations of FTs of two volumes, with the second one being conjugated. Since in our case volumes are real, their FTs have Friedel symmetry, *i.e.* for 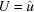 and for all elements indexed by **s**, we have *U*(**s**) = *U*(−**s**)*. Therefore, the result of UV* is a scalar (a real number), as for each complex product of elements there exists a Friedel-related conjugated complex product. This also holds for the calculation of inner products within Fourier ‘shells’. Therefore, to make it transparent, the inner product of the Friedel symmetric complex vectors is often written as 2Re(UV*).

### 2.2. Modified Fourier shell correlation (mFSC)   

We begin by noting that the signal-to-noise ratio (SNR) in the image, defined as the ratio of the variance of the signal to the variance of the noise in the data, can be equivalently estimated either in real or reciprocal space. Indeed, given two zero-mean half-volumes *u* and *v* and their Fourier transforms 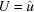 and 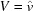, the correlation coefficient is
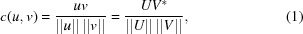
which is a normalized inner product of two vectors and has values in the interval (−1, 1), with both extremes indicating perfect agreement, while zero indicates no relation between arguments. The correlation coefficient is related to the SNR by (Saxton, 1978 ▸)
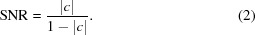
Since by definition the SNR cannot be negative, we used the absolute value of the correlation coefficient. This agrees with the intuitive notion that *c* = −1 means that both volumes are identical, albeit with opposite contrast (note that this cannot happen in the standard practice of cryo-EM). Another possibility would be to have SNR = 0 for *c* < 0. The choice of either possibility is irrelevant for the purpose of the following. What matters is that the values of the correlation coefficient (or FSC, as will be shown) map nonlinearly to the SNR. The value *c* = 0 means that there is no shared signal in the half-volumes, while *c* = 1 means that the SNR is infinite, which implies that the volumes are noise-free. Of interest are intermediate values; for example, *c* = 0.143 yields an SNR of merely 0.17 and thus a signal over five times lower than the noise. *c* = 0.5 yields SNR = 1, which means that the amplitudes of the signal are of the same magnitude as those of the noise. Only for *c* = 0.91 do we have SNR = 10, a level of signal that is comfortably above the level of noise.

The relationships between (1) and (2) form the basis for the FSC methodology. The SNR given by (2) informs us about the quality of the data within the entire real-space window within which the structure is computed and for all spatial frequencies simultaneously. The goal is to restrict calculations of the correlation to a certain region in order to obtain a value that is ‘localized’ in real or Fourier space or preferably in both spaces at the same time. So, for FSC calculations we chose a sequence of nonoverlapping Fourier space rectangular spherically symmetric concentric window (binary) functions *W*, referred to as ‘shells’, with preselected width (usually set to one Fourier pixel). These shells in 3D are centered on the origin of Fourier space and have radii corresponding to the sequence of magnitudes of spatial frequency *s* = ||**s**||,
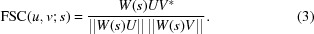
For each shell, the spectral SNR (SSNR) at spatial frequency *s* is given by
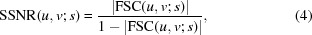
where a semicolon separates groups of variables. While both the FSC and the SSNR are functions of both half-volumes and spatial frequency (they are also a function of shell width, which we have omitted for simplicity), we separate variables to indicate that for typical calculations we consider half-volumes to be fixed and we only analyze the FSC (and the SSNR) as a function of spatial frequency. We also note that as elaborated in Section 2.1, the outcome of the numerator calculation in (3) is real, and so is the FSC.

For the vast majority of practical applications it is necessary to restrict the real-space region of support to the area *m* occupied by the structure of a complex, and thus to exclude surrounding noise. *m* is a real-space function of the same size as the half-volume and, in the ideal case, is binary, *i.e.* contains only ones that indicate the structure and zeroes elsewhere:
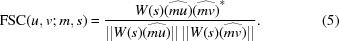
Here we treat the mask as a variable, as in later parts of the text we show results in which both *m* and *s* vary. The calculation of (5) calls for the multiplication of both half-volumes by the mask, followed by the computation of FTs, and finally calculation, in Fourier space and within shells defined by the window function, of the inner product of the processed half-volumes as well as the norms. The result is the FSC, a one-dimensional real-valued function of spatial frequency that informs us how well two volumes agree in Fourier space. The decrease of the FSC below a predefined threshold is called the frequency limit or resolution of the structure represented by the half-volumes.

From (5),[Disp-formula fd5] it is immediately apparent that the outcome of this simple procedure is bound to be marred by artifacts. The problem is owing to the real-space mask entering the calculation prior to the FT of the maps *u* and *v*. Firstly, it is intuitively clear that even if the half-volumes were two realizations of independent random noise, and thus the expected value of the FSC should be zero for the entire frequency range, the multiplication of both by the same mask function *m* introduces a ‘common’ signal, that is the shape of the mask, which results in significant FSC coefficients. The more elaborate the mask, the stronger the effect. On a more fundamental level, we consider the convolution theorem, which states that the FT of a product of two functions is a convolution of their FTs, and this holds for the product of real as well as complex functions. Therefore, the multiplication of a real half-volume by a mask followed by an FT, as in the right-hand side of (5), is equivalent to the convolution of the FT of the half-volume with the FT of the mask. Such a convolution introduces correlations between the coefficients of the FT of a half-volume, and as a result superfluous correlations and artificially high FSC values.

To solve the problem of the mask-induced artifacts and thus incorrect estimates of resolution by the FSC technique, we can change the order of calculations in (5) and have 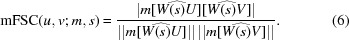
The modified FSC (mFSC) is computed in real space. Firstly, the FTs of both maps are multiplied by the reciprocal-space window *W*, retaining only information within a shell with a radius of the magnitude of spatial frequency *s*. Secondly, after inverse FTs, the real-space correlation coefficient is computed within a region defined by the real-space mask *m*. The second operation simply restricts the number of elements used for calculation and thus cannot induce any spurious correlations. In order to reduce ringing artifacts (Gibbs phenomenon) induced by the truncation of the Fourier expansion by the rectangular window *W*, we instead use a Gaussian bandpass filter with a preselected width (we usually set its standard deviation to one Fourier pixel). Admittedly, this diffuses the localization of the mFSC in reciprocal space but focuses the resolution measure in real space. This is an unavoidable trade-off between localization in the two respective spaces inherent to the finite Fourier transform of a finite series. One also has to note that the computational complexity of the mFSC is enormously higher than that of the FSC, as in the latter, reciprocal-space approach only two 3D FTs have to be computed, while the mFSC requires two 3D FTs for each Fourier shell *s*. While the specific wall-clock time depends on the details of the implementation and computer used, the FSC can be calculated within seconds, while the mFSC requires minutes of calculation time. However, if a message-passing interface (MPI) parallelized implementation is used, given that the algorithm is trivially parallelizable, the time of the mFSC calculation is reduced by the number of cores used and for large clusters corresponds to that for the FSC.

### 2.3. Local resolution estimation with the mFSC   

Local resolution calculation follows trivially from the concept of the mFSC: we compute a local real-space mFSC by evaluating the correlation coefficient (4) within a small region *m*(**x**) (say a box of 15 × 15 × 15 voxels, or a small-radius sphere) in real space at all real-space locations **x**,
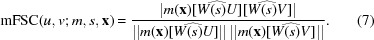
We present the outcome of the local resolution calculation as a 3D volume whose voxel values are local spatial frequencies at which the local resolution drops below a predetermined mFSC threshold level *t*, for example *t* = 0.5 or *t* = 0.143. In addition to the direct interpretation in the familiar FSC or SSNR terms, the volume provides the necessary input for the local filtration step.

We implemented local resolution estimation based on the mFSC concept espoused previously (Penczek, 2014*b* ▸), but we found the quality of the results to be lacking. Without estimation of the ndf and with the small local window sizes used the results were unstable, noisy and difficult to interpret. To somewhat improve the results we used *t* = 0.5, but this did not eliminate the noise problem entirely. The current implementation is based on adjustment of the local window size based both on the ndf and on the desired smoothness of the local mFSC curve.

### 2.4. Estimation of the number of degrees of freedom (ndf) in single-particle maps   

The resolution estimates discussed here involve the calculation of correlation coefficients. Normally, a threshold is preselected based on a more-or-less convincing rationale and a point is identified at which the resolution curve decreases below this threshold. The inverse of the spatial frequency at which this happens is called the ‘resolution’. A more sophisticated approach calls for a statistical test, *i.e.* the determination of a point at which the resolution curve is still significantly higher than the preselected threshold. In this case one also has to choose the significance level; thus, such an approach requires the selection of values for two arbitrary parameters. Tests of the correlation-coefficient significance exist and require only mild assumptions about the underlying statistical distributions. However, they do require the noise in the data to be approximately Gaussian and call for the determination of the number of degrees of freedom (ndf). This constitutes a challenge which until now has not been satisfactorily addressed.

For many problems in image processing one can either ignore the exact nature of the relations between samples or at least assume that the assumption of the normality of noise distribution is not severely violated. It is then reasonable to argue that the ndf should be close to the number of samples. However, in cryo-EM this is far from true as image formation in electron microscopy (modeled by the contrast transfer function and envelope functions; Zhu *et al.*, 1997 ▸) and the final stages of single-particle analysis, such as the calculation of 2D class averages or the 3D reconstruction of maps which involve interpolation, Fourier filtration and masking of the data, introduce pixel-wise correlations. As a result, the ndf is expected to be significantly lower than the number of samples.

A standard way to make the noise component in the data have the statistical properties of white noise is to apply a ‘prewhitening’ step, *i.e.* to divide the Fourier transform (FT) of the signal by the FT amplitudes. This makes the power spectrum flat and the autocorrelation function close to a delta function, as expected for white noise. However, this does not necessarily mean that the Fourier components are statistically independent, as prewhitening in reciprocal space does not result in a stationary distribution of noise amplitudes in real space. Any unevenness in real space means there is a residual convolution of Fourier components and thus the ndf is lower than the number of samples.

In the following, we will demonstrate that in the case of cryo-EM single-particle reconstruction prewhitening is sufficient to accomplish a degree of independence of noise components; moreover, the step is implied in the FSC/mFSC methodology. We next show that the reduction of the ndf owing to the Gaussian window *W* can be accurately accomplished using principal component analysis (PCA). Finally, given that the mFSC uses only a binary mask, the resulting adjustment of the ndf is obtained in a straightforward manner by taking into account the fraction of the entire volume occupied by the mask.

Given the two half-maps *u* and *v* with the image-formation model
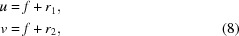
where *f* is the shared signal and *r*_1_ and *r*_2_ are independent realizations of random noise, we estimate the residual noise in the determined structure as
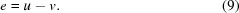
The prewhitening simply requires the division of the FT of an image by the rotational average of the square root of its power spectrum, resulting in an image whose power spectrum is flat and constant. In Fourier space we will write this as
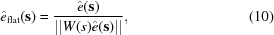
where the window function *W*(*s*) has the same meaning as in (3), *i.e.* it is a binary ‘shell’, and, for each spatial frequency *s*, the denominator is a norm within such a shell. The subscript ‘flat’ denotes the result of prewhitening; that is, an image with a flat power spectrum. We recognize that the right-hand side of (10) corresponds to those of (5) and (6), *i.e.* the computation of both the FSC and the mFSC involves normalization within spatial frequency shells, therefore the prewhitening is implied. While it cannot be proven in general, for the cryo-EM structures that we tested the autocorrelation function of *e*_flat_(**x**) is very narrow (below one pixel). More importantly, while the rotational average (in real space) of *e*(**x**) has a distinct profile (as expected, the solvent noise level external to the structure is higher than the noise within the region of the structure), the rotational average of *e*_flat_(**x**) is reasonably flat (Supplementary Fig. S1). Therefore, we conclude that owing to the way that the FSC (and mFSC) are computed, the implied prewhitening is sufficient and no explicit step is needed. Finally, this implies that the overall ndf ≅ *n* ^3^, where *n* is the linear size of the reconstruction volume in voxels.

It follows that in the simple case of the FSC and in the absence of a real-space mask, the ndf is given by the number of Fourier components included in the calculation. Assuming a shell width of one pixel, we have 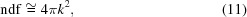
where we have replaced the spatial frequency *s* by an integer index *k* of a Fourier transform pixel, with the two being related by *s* = *k*/*n*, *k* = 0, 1, …, *n*/2. The latter is more convenient to use in the context of ndf analysis.

For the mFSC we use a Gaussian window, and in this case an exact calculation of the ndf is not straightforward. Therefore, we used principal component analysis (PCA) to determine the number of independent components by using an array of random noise multiplied by a Gaussian window function. The Monte Carlo simulation procedure comprises the following steps.

(i) Initialize.
  (1) Set the array length *n*.
  (2) Initialize the covariance matrix *c*(*n*, *n*) = 0 and a shuffled covariance matrix *d*(*n*, *n*) = 0.
  (3) Set the number of Monte Carlo procedure iterations.
(ii) Generate an array of *n* independent randomly distributed samples from a Gaussian distribution *N*(0, 1).
(iii) Multiply the array by a Gaussian window function *w*_g_(*k*).
(iv) Compute the outer product of the array and add it to the covariance matrix *c*.
(v) Shuffle the order of the entries in the array, compute the outer product of the outcome and add it to the shuffled covariance matrix *d*.
(vi) Repeat steps (ii)–(v) a preset number of times.
(vii) Perform eigenanalysis of the covariance matrices *c* and *d*.
(viii) Find the highest index *c*-matrix eigenvalue that is larger than the corresponding *d*-matrix eigenvalue.

In step (v) of the procedure we incorporated the randomization test for the number of principal components that sufficiently represent the data set. Briefly, randomization (‘shuffling’) of the order of the entries results in a data set that no longer has any common structure. However, the resulting pure noise data set has the same noise properties as the original, nonrandomized set and can serve as a control. Indeed, we retain only those components of the original data set whose eigenvalues are larger than the corresponding eigenvalues of the shuffled set.

The eigenvalue index found in step (viii) is the effective ndf associated with a Gaussian window function *w*_g_(σ_g_). We found that to a good approximation ndf ≅ 3σ_g_ (Supplementary Fig. S2). It follows that for a 3D volume we have
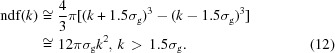
The ndf is a quadratic function of the spatial frequency index *k* and for typical volume sizes used in cryo-EM its values range from ndf(5) ≅ 1000 to ndf(200) ≅ 1 500 000 (Supplementary Fig. S2).

(12) gives ndf values in the absence of any masking during mFSC calculation. In all practical applications the use of a mask is a necessity as otherwise the resolution will be underestimated owing to the presence of noise surrounding the structure. It is straightforward to notice that for a real-space mask with |*m*| nonzero elements, (12) has to be modified to reflect the fact that only a fraction of |*m*|/*n* ^3^ real-space voxels is included in the calculations:
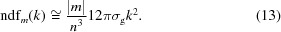
We note that molecules typically occupy a relatively small region of the volume. The reasons for this are (i) imperfect particle picking, meaning that the center of the projected molecule can be far from the center of the 2D window, so as a precaution window size has to be considerably larger than particle size, (ii) the necessity to represent the contrast transfer function (CTF) faithfully (Penczek *et al.*, 2014 ▸) and (iii) the need to avoid artifacts induced by reciprocal-space operations in the discrete domain. It is often suggested that the window size should be about 30% larger than the diameter of the molecule. A spherical mask with a diameter of 0.7*n* voxels thus occupies merely 18% of the volume. For high-resolution structures and masks that closely follow the shape of the molecule, given the presence of many invaginations and openings, the occupied space can be less than 10% of the volume.

### 2.5. mFSC one-sided confidence interval   

We previously showed that using Fisher’s *z*-transformation of a correlation coefficient and the associated ndf, it is straightforward to compute two-sided confidence intervals (CIs) for a given value of FSC (Penczek, 2010 ▸). Further, we showed that asymptotically, for FSC = 0 and at a confidence level of 95% (statistical significance α = 5%), CIs correspond to the 3σ criterion introduced over three decades ago. This is however incorrect, as the FSC/mFSC does not oscillate wildly (unless serious data-processing mistakes were made); instead, in all practical situations it decreases quasi-monotonically from a value of one at low spatial frequencies (structures are computed from random halves of the same data set, so at the very least they are expected to have the same overall shape) to zero beyond the resolution limit of the structure (in the higher spatial frequency region, in which the signal is entirely dominated by noise). We are instead interested in the spatial frequency at which the FSC/mFSC is no longer higher than some resolution cutoff threshold *t*. The inverse of the spatial frequency at which this occurs we call the resolution of the structure. Therefore, a sensible approach is to select *t*, the confidence level, or equivalently to select statistical significance α as the criterion and compute the one-sided CI. The use of a CI has two major advantages: (i) the test incorporates information about the statistical distribution of the FSC/mFSC (associated with the error of measurement) and (ii) it includes the number of degrees of freedom (effective sample size), thus preventing the acceptance of results computed from a small number of elements, which are unreliable. The latter point is of particular importance for cryo-EM, where it is easy to notice that the smaller the area of the mask, the ‘better’ the resolution results, if read from the FSC curve directly.

We use a *z*-transformation of the mFSC, 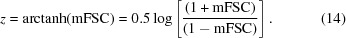
For a number of samples larger than 25 and mFSC < 0.95, *z* is approximately normally distributed with variance 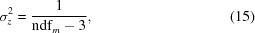
and the one-sided upper confidence interval is
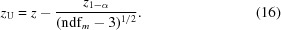
(Note that to obtain the two-sided CI, α should be replaced by α/2.) Finally, *z*_U_ has to be transformed back to obtain a one-sided upper CI for the mFSC,
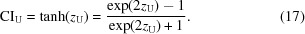
For each spatial frequency index *k*, we check the condition
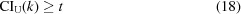
and we select the highest *s* for which it remains fulfilled. We then set the resolution to *n*/*k* pixels, or given a pixel size *p* Å, to *pn*/*k* Å.

We first note that for large ndf_*m*_, CI_U_ is a slowly decreasing function. This justifies our approach for the estimation of ndf_*m*_, as given that in general we consider only two significant digits of mFSC (the accuracy limit is predominantly imposed by the size of a reciprocal-space pixel, *i.e.* the sampling of the mFSC curve), such an approximate value of ndf_*m*_ is entirely sufficient to obtain a sensible confidence interval. Secondly, the two-sided CI for the correlation coefficient can be related to the standard deviation of the *z*-transformed coefficient and α = 5% corresponds to 2σ. However, the 3σ criterion was probably inspired by the use of more stringent standards in fields where data are well behaved, as it corresponds to the statistical significance α = 0.3%, a level that is excessive in biological statistical data analysis. It is also likely that it was chosen to compensate for the overestimation of the ndf prevalent at the time in 3D cryo-EM structure determination. For a one-sided CI scaling the use of units of standard deviations is inappropriate as the test is asymmetric. Indeed, the upper interval is 
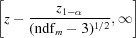
and replacing *z*_1−α_ by the number of standard deviations is not informative. Moreover, for 2σ we have α = 2.3%, while for 3σ we have α = 0.1%, which is excessive. For these reasons we scale CI_U_ in significance levels and give preference to α = 1%. Admittedly, the choice of statistical significance depends on the data and ultimately it is the decision of the researcher.

## 3. Results   

### 3.1. Single-particle reconstruction resolution estimation   

For tests of single-particle resolution estimation by the mFSC, we used the data set for the *Plasmodium falciparum* 80S ribosome bound to the antiprotozoan drug emetine deposited in the EMPIAR archive as entry EMPIAR-10028. The data set comprised 105 247 single-particle projection images, with a window size of 360 × 360 pixels and a pixel size of 1.34 Å on the specimen scale (for the remaining details, see Wong *et al.*, 2014 ▸). The diameter of the complex was ∼295 Å or 220 pixels. The 3D refinement was performed using the ML-based program *sxmeridien.py* implemented in *SPARX* (Hohn *et al.*, 2007 ▸). The nominal resolution using a soft mask (the same as used in the refinement) was 3.24 Å at 0.143 cutoff FSC (149 Fourier pixels). All resolutions given in the following and in the figure legends are obtained using a threshold of *t* = 0.143, unless otherwise noted.

**Figure 1:**
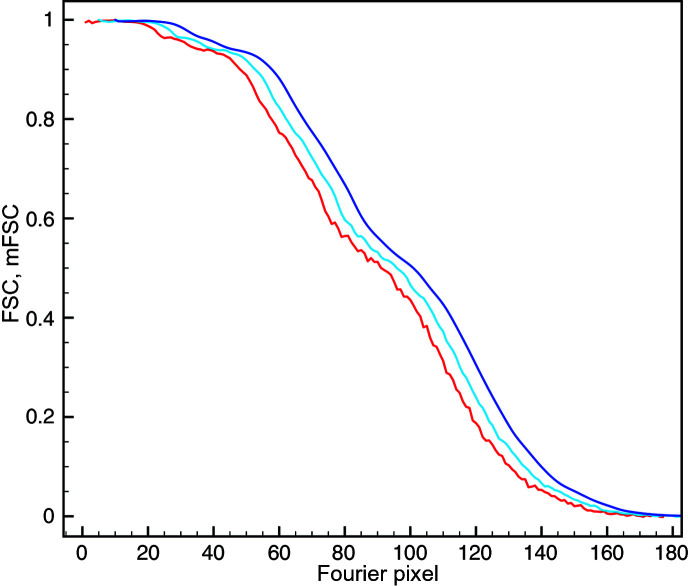
The reciprocal-space window is responsible for the smoothness of FSC and mFSC curves. Red: FSC curve, shell width one Fourier pixel. Light blue: mFSC, σ_g_ = 1. Dark blue: mFSC, σ_g_ = 3. All three curves coincide, so for the purpose of illustrating their shape they were shifted along the *x* axis by five pixels with respect to each other.

We first observe that in the absence of a mask the FSC and mFSC results coincide, as in this case (5) and (6) are equivalent. The only difference is the reciprocal-space window function, which in the former case is rectangular and in the latter is a Gaussian function. In the absence of a mask, the FSC and mFSC curves coincide, but owing to the use of Gaussian function the mFSC curves are smoother (Fig. 1 ▸). The smoothness of the mFSC curves is proportional to the value of the half-width σ_g_. At the same time, while σ_g_ = 3 may yield visually more appealing results, each point of the curve represents a broader range of resolution values than for σ_g_ = 1, so the curve is less detailed.

**Figure 2:**
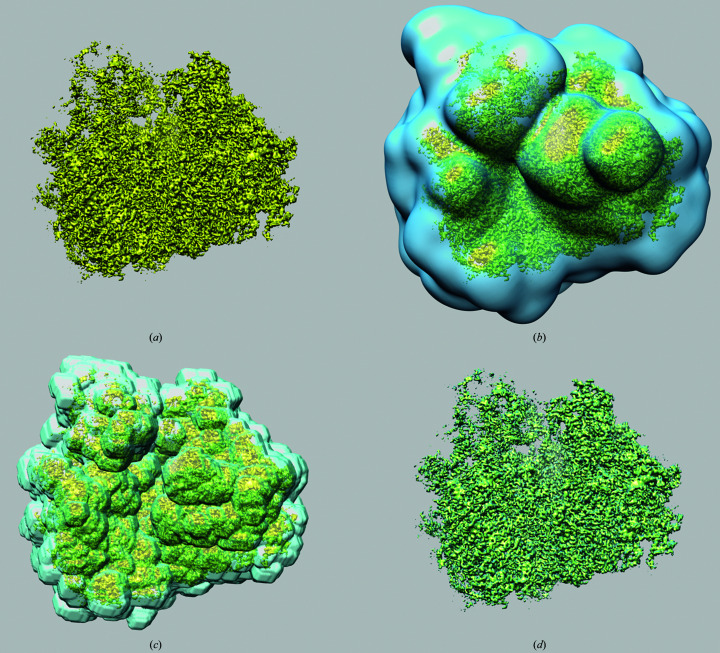
3D reconstruction of the 80S ribosome (data set EMPIAR-10028). (*a*) Surface representation of the low-pass-filtered ribosome after power-spectrum adjustment with a Gaussian high-pass filter. The structure has multiple flexible surface components and there is a residual ratchet movement of the 40S subunit (the left part of the structure in the orientation shown). (*b*) A generous mask used during structure refinement fills 11.5% of the volume. (*c*) A tight mask that typically would be used for resolution assessment fills 6.5% of the volume. (*d*) A very tight mask following the outline of the structure and eliminating the most flexible regions and any solvent noise fills 0.6% of the volume.

Of main concern in resolution estimation is the influence of a real-space mask. To test the comparative performance of the FSC and mFSC, we generated a series of three smooth-edged masks with a cosine fall-off, with edges extending to five pixels (for use with FSC, and after binarization at a threshold of 0.5 for use with mFSC; Fig. 2 ▸). The first, generous mask was used in the 3D refinement. Since this particular 80S sample had previously been documented to have extensive conformational variability (Wong *et al.*, 2014 ▸), to avoid the suppression of any peripheral and poorly resolved structural details this mask was much broader than one would normally use in refinement. The second mask was created in order to assess the final resolution of the 80S map and closely follows the overall shape of the ribosome. The third mask was created to assess the influence of solvent within the structure and thus it was generated using the surface threshold selected to display the structure, at which the solvent is not visible. All mFSC curves were computed with σ_g_ = 1 and a one-sided confidence interval (CI) at α = 1%.

**Figure 3:**
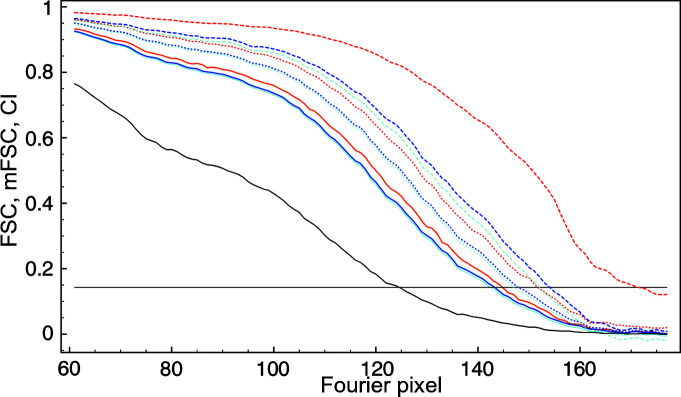
The influence of a mask on FSC and mFSC resolution estimation. Black: mFSC without a mask; resolution 124 pixels (3.89 Å), CI (not shown) 123 pixels (3.92 Å). Solid red: FSC using a generous mask [Fig. 2 ▸(*b*)]; resolution 144 pixels (3.35 Å). Solid blue: mFSC using a generous mask [Fig. 2 ▸(*b*)]; resolution 143 pixels (3.37 Å). Solid light blue: CI of the mFSC; resolution 142 pixels (3.40 Å). Dotted red: FSC using a tight mask [Fig. 2 ▸(*c*)]; resolution 151 pixels (3.19 Å). Dotted blue: mFSC using a tight mask [Fig. 2 ▸(*c*)]; resolution 147 pixels (3.28 Å). Dotted light blue: CI of the mFSC; resolution 147 pixels (3.28 Å). Dashed red: FSC using a very tight mask [Fig. 2 ▸(*d*)]; resolution 171 pixels (2.82 Å). Dashed blue: mFSC using a very tight mask [Fig. 2 ▸(*d*)]; resolution 154 pixels (3.13 Å). Dashed light blue: CI of the mFSC; resolution 152 pixels (3.17 Å). The horizontal line marks the *t* = 0.143 resolution-cutoff threshold. All mFSC curves were computed with σ_g_ = 1 and one-sided confidence interval (CI) at α = 1%.

In the absence of a mask the CI resolution was 123 pixels (3.92 Å), and it was only one Fourier pixel less than the mFSC resolution (Fig. 3 ▸). Similarly small differences between the resolution values occurred in the case of a generous mask: FSC yielded 144 pixels (3.35 Å), while the mFSC CI resolution was 142 pixels (3.40 Å). This is expected as the ndf in the latter case was very large. Although this mask occupied only 11.5% of the volume, the ndf at *t* = 0.143 was 88 000. For the tight mask, which was used to yield the ‘reported’ resolution, FSC yielded 151 pixels (3.19 Å), while the mFSC CI gave 147 pixels (3.28 Å), which is significantly lower and attests to the exaggerated resolution estimates obtained with the traditional FSC, even though this mask remains conservative by currently acceptable standards. The mFSC estimate is robust even though the tight mask occupies only 6.5% of the volume. The solvent-excluding mask was very tight and essentially coincided with the displayed structure [Fig. 2 ▸(*d*)]. As expected, the FSC was artifactual as it never reached the zero level and yielded an unrealistic resolution of 171 pixels (2.82 Å), which can be dismissed by visual examination of secondary elements within the map. However, the mFSC CI yielded a resolution of 152 pixels (3.17 Å), which is only two Fourier pixels or 0.11 Å better than that for the tight mask, a difference which arguably would not change the visual appearance of the map if used for final low-pass filtration. Moreover, the curve is correct as it decreases to zero at very high frequencies despite the fact that the mask occupies only 0.6% of the volume, and at *t* = 0.143 the ndf was 11 700. This proves that the mFSC CI is a proper and robust resolution measure that is immune to the influence of the mask.

### 3.2. Helical reconstruction resolution estimation   

The introduction of the iterative helical real-space reconstruction (IHRSR) protocol two decades ago revolutionized the structural determination of helical assemblies (Egelman, 2000 ▸). It employs a modified single-particle projection-matching approach (Penczek *et al.*, 1994 ▸), and by using relatively short, consecutive filament segments made it possible to process specimens that resisted analysis with the previously dominant diffraction-based approach to high resolution (Diaz *et al.*, 2010 ▸). Indeed, the use of short segments alleviates the problems caused by the natural flexibility of helical assemblies, the wobbling of helical symmetry parameters and also structural heterogeneity (Egelman, 2015 ▸). However, estimation of the resolution by the FSC has so far proved to be unreliable and no sensible approach has been put forward. This is owing to a combination of factors: (i) helical structures can be thought of as a sequence of ‘unique’ disks (a concept similar to asymmetric subunits in point-group symmetries) that are rotated and stacked in the *z* direction. It is all but impossible to isolate one using a classical FSC mask approach without inducing severe artifacts. In many cases these disks are very short, barely exceeding a few pixels. The inclusion of more than one within a mask induces spurious correlations owing to helical relations between them. Finally, a helical structure is infinite from a mathematical point of view and has to be truncated to fit a given box size, the dimensions of which only rarely coincide with integer multiplicity of unique disks, thus introducing undesirable relations between the Fourier transform components. In the following we will show that the mFSC approach all but eliminates all of these obstacles and yields a reliable measure of the resolution of helical assemblies.

**Figure 4:**
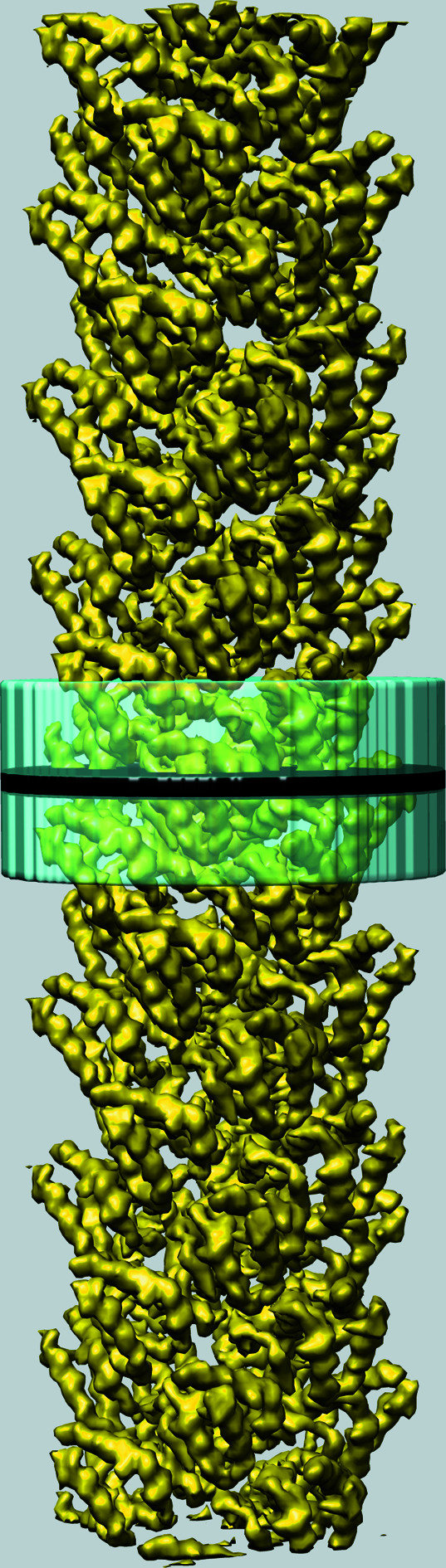
Cryo-EM structure of the CARD^MAVS^ filament. Black: a single ‘unique’ disk. Transparent blue: the cylindrical mask used for mFSC calculation. Its height is eight ‘unique’ disks.

For tests of helical resolution estimation by the mFSC we used the CARD^MAVS^ filament data set (Wu *et al.*, 2014 ▸). The published structure was determined using the *HELICON* helical processing suite of programs (Penczek, 2014*a* ▸), which employs a modified IHRSR strategy and is implemented in * SPARX* (Hohn *et al.*, 2007 ▸). The data set comprised 12 817 projection images of filament segments, with a window size of 256 × 256 pixels and a pixel size of 1.24 Å on the specimen scale. The radius of the filament was 46 Å (or 37 pixels). The axial rise per asymmetric unit in the filament was 5.13 Å and the azimuthal rotation per subunit was 101.15° (for the remaining details, see Wu *et al.*, 2014 ▸). It follows that the ‘unique disk’ was 5.13/1.24 ≅ 4.14 pixels in height. In order to minimize the problem with fractional disk size, we created a cylindrical mask with a radius of 37 pixels and of 4.14 × 8 = 33.12 ≅ 33 pixels in height (Fig. 4 ▸). During mFSC calculations we adjusted the estimated ndf by dividing it by the number of included disks; that is, eight.

**Figure 5:**
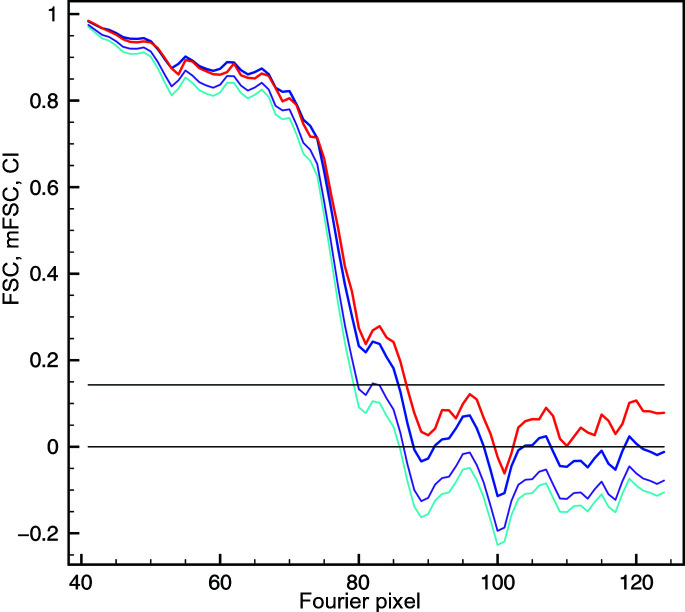
Resolution estimation for the CARD^MAVS^ filament structure. Red: FSC with a cylindrical mask, height 256 pixels (entire volume), radius 37 pixels; resolution 86 pixels (3.69 Å). Dark blue: mFSC with a cylindrical mask, height 80 pixels (eight ‘unique’ disks), radius 37 pixels; resolution 85 pixels (3.73 Å). Magenta: CI at a 5% significance level; resolution 79 pixels (4.02 Å). Light blue: CI at a 1% significance level; in this case the resolution is the same as for the 5% CI.

The FSC resolution of the CARD^MAVS^ filament structure was 86 pixels (3.69 Å). The resolution curve was however artifactual, as while it did intersect the zero threshold, it subsequently rose and oscillated, adopting only positive values (Fig. 5 ▸). Conversely, the mFSC CI resolution based on the eight-disk region was 79 pixels (4.02 Å). Not only is the curve correct, oscillating about the zero level at very high frequencies,^1^ but the resolution obtained appears to better correspond to the visual appearance of the secondary elements in the structure. This demonstrates that the mFSC is a proper tool for bias-free resolution estimation of helical assemblies determined by the quasi-single-particle approach of IHRSR.

### 3.3. Local resolution estimation   

For tests of local resolution estimation in single-particle maps by the mFSC, we used the data set for the *P. falciparum* 80S ribosome described in Section 3.1. In order to find conditions that would reduce the noisiness of the outcome, we performed a number of trials. We computed a set of mFSC curves for box sizes of 11^3^, 13^3^ and 15^3^, and set the box location within the ribosome map and varied it within ±1 pixel. We also set the Gaussian window width to σ_g_ = 3 and the one-sided confidence interval (CI) to α = 1%. We compared resolution determination at *t* = 0.143 and we determined that an acceptable dispersion of values between neighboring box locations was for a box size of 15^3^ (results not shown). Moreover, the smaller window size of 11^3^ reduces the ndf in comparison with a box of 15^3^ about 2.5 times; thus, the resolution estimates are accordingly lower and are below the overall resolution estimate for the 80S ribosome structure.

**Figure 6:**
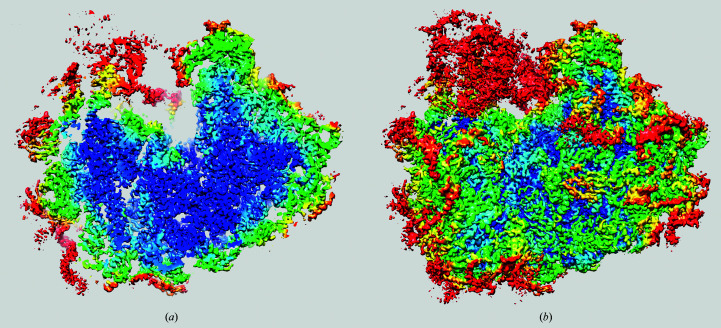
Local resolution map of the 80S ribosome. (*a*) Side view: left, 40S subunit; right, 60S subunit. (*b*) Central section of the resolution map in the orientation shown in (*a*). The resolution is color coded as follows: red, 3.69 Å; yellow, 3.57 Å; green, 3.35 Å; light blue, 3.15 Å; dark blue, 2.98 Å.

The local resolution map is shown at five resolution values from 3.69 to 2.98 Å, with the highest resolution value present in the map being 2.79 Å [Fig. 6 ▸(*a*)]. The average value of the local resolution within the map region was 3.26 Å. The overall resolution of the map computed with a tight mask [Fig. 2 ▸(*c*)] and σ_g_ = 3 was 3.24 Å, which demonstrates excellent agreement between the overall mFSC estimate and the local resolution mFSC estimate. The resolution map is highly detailed and supports the previous interpretation of the *P. falciparum* 80S ribosome structure. The peripheral elements are either flexible or substochiometric and thus have lower resolution, the head of the small 40S subunit swivels, and internal parts of the structure are more rigid and have higher resolution.

Of concern is the appearance of the central section of the local resolution 80S map [Fig. 6 ▸(*b*)]. It is striking that the local resolution has a pronounced radial fall-off, *i.e.* the central part has high resolution and it becomes progressively worse towards the outer part of the complex. There are various possibilities why this is the case. (i) It reflects the actual properties of the complex. This is however unlikely as the radial dependence is too regular to be accidental and the same effect has been observed in other results published by us and others. There seems to be growing awareness that the variability of external parts of cryo-EM complexes is likely to be owing to data-processing artifacts (Liu *et al.*, 2018 ▸). (ii) The effect is owing to a combination of sample properties and the way that the 3D refinement was carried out. Indeed, most ribosome samples contain a combination of various states. In particular, the presence of the so-called ratchet rearrangement of subunits is often observed (Frank & Agrawal, 2000 ▸; Ratje *et al.*, 2010 ▸). This is a relative rotation of small (40S) and large (60S) subunits with respect to each other. As a result, in local resolution maps all peripheral elements will appear to have a lower resolution, but this is simply an artifact induced by the choice of system of coordinates. This was noted and counteracted by the development of a dedicated refinement procedure in which multi-particle 3D sorting was combined with focusing 3D refinement of the large ribosomal subunit (Penczek *et al.*, 2014 ▸; Behrmann *et al.*, 2015 ▸). While this explains at least part of the strong resolution dependence in the case of ribosome samples, it does not explain the similar dependence observed for other systems. (iii) Finally, the radial dependence might be to a large extent a trivial reflection of the distribution of 3D alignment errors. The alignment involves two estimates for each 2D projection image: firstly the in-plane translation, which is believed to have small errors and which moreover would not manifest itself in a radial fashion in 3D, and secondly angular errors, as 3D projection images are oriented in polar coordinates and three Eulerian angles have to be established. These rotational errors are typically larger than the translational errors and, needless to say, would exhibit a strong radial dependence in local resolution maps.

### 3.4. Segment-focused resolution estimation   

**Figure 7:**
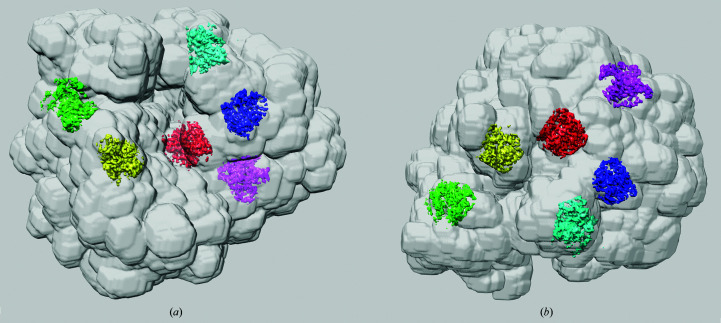
Resolution of six selected segments (out of 100) extracted from the 80S map. (*a*) Side view. (*b*) Top view. In both views the small subunit (40S) is on the left and the large subunit (60S) is on the right. The resolution was estimated using σ_g_ = 3, CI at a 1% significance level and *t* = 0.143. The overall resolution of the 80S ribosome with the very tight mask was 156 pixels (3.09 Å). Segments: green, 126 pixels (3.83 Å); yellow, 154 pixels (3.13 Å); red, 158 pixels (3.05 Å); light blue, 144 pixels (3.35 Å); dark blue, 152 pixels (3.17 Å); magenta, 141 pixels (3.42 Å).

We extracted 100 segments from the 80S map using the *e2segment3d.py* utility, *k*-means option, as available in *EMAN*2 (Baker *et al.*, 2012 ▸). This roughly corresponds to the number of component proteins in the eukaryotic ribosome, which is 79–80. From these, we chose six such that they would represent various regions of the 80S ribosome (Fig. 7 ▸). We made no attempt to make sure that the extracted segments are biologically meaningful, as this would require expertise and effort far exceeding the scope and purpose of this work. *e2segment3d.py* extracted segments as binary masks, so we used them directly to compute, independently for each of them, segment-focused mFSCs using σ_g_ = 3, CI at a 1% significance level and *t* = 0.143.

**Figure 8:**
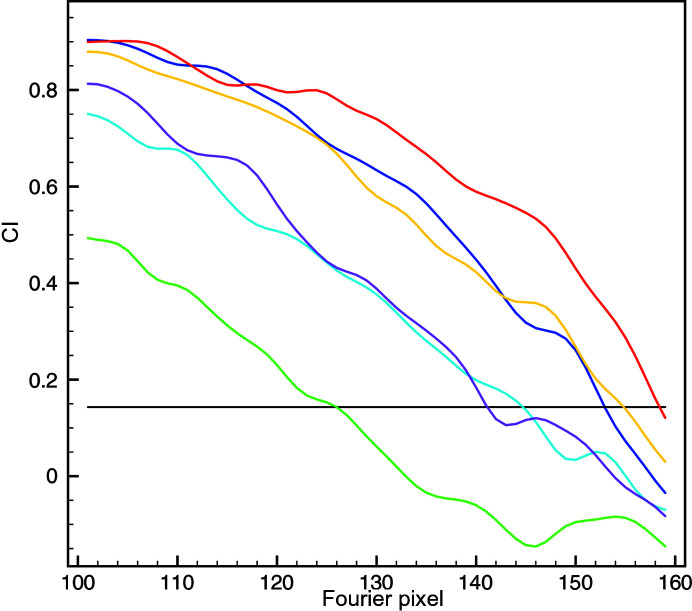
Resolution CI curves at a 1% significance level of six selected segments (out of 100) extracted from the 80S map. The resolution was estimated using σ_g_ = 3, CI at a 1% significance level and *t* = 0.143. The color coding of the curves matches the color coding of the segments in Fig. 7 ▸.

The resolution of the entire 80S ribosome using the very tight mask (which follows the structure closely and thus best corresponds to the results of segmentation) and σ_g_ = 3 was 156 pixels (3.09 Å). The resolution of segments varied between 126 pixels (3.83 Å) and 158 pixels (3.05 Å) (Fig. 8 ▸). These results generally agree with those of local resolution based on the moving box: peripheral segments (green, light blue, magenta) that are located close to the external surface of the ribosome have a lower resolution, while those buried inside (red, dark blue) have a higher resolution.

The segment-based mFSC yields results that are easier to interpret as it eliminates the problem of gradual changes in local resolution, which stem from the limited spatial resolvability of the box-based method. The method also allows one to probe the resolution of functionally relevant subunits of a structure or even secondary-structure elements such as larger α-helices or β-sheets. We also note that on average each segment occupies 0.008% of the volume, which is about the same as the 15^3^ box used in the local resolution in Section 3.2. However, the masks of the segments only include protein (or RNA in the case of ribosome), while the boxes contain a large percentage of solvent areas, so segment-focused mFSC yields a more reliable assessment of the local resolution of the structure.

## 4. Implementation   

The methodology described here was implemented in the *SPARX* system (Hohn *et al.*, 2007 ▸) as the *sxresolution* command (Penczek, 2020 ▸). *SPARX* is distributed jointly with the *EMAN*2 system (Tang *et al.*, 2007 ▸). The program was written in high-level Python3, with the CPU-intensive components written in low-level C++. Statistical special functions are computed with *SciPy* (Oliphant, 2007 ▸). The 3D Fourier transforms are computed using the FFTW3 library (Frigo & Johnson, 2005 ▸). Since the most time-consuming part of the execution of the code is the calculation of the 3D FTs, we used a threaded version of FFTW3.

## 5. Discussion   

We have addressed two fundamental issues in resolution estimation of cryo-EM maps using the FSC methodology: (i) the strong influence of a mask on the results and (ii) the estimation of the number of degrees of freedom (ndf) in the data, which allowed us to properly calculate one-sided confidence intervals (CIs) for FSC coefficients. We termed the new method modified FSC (mFSC) and we stress the fact that it affords the user freedom to choose almost any mask. We showed that not only does the mFSC yield a reliable map of local resolution values, but also segment-focused resolution curves, and thus it permits quality exploration of functionally significant regions, segments and even larger secondary elements of cryo-EM maps.

We demonstrated that the mFSC is free of mask-induced artifacts. However, we determine the resolution not based on the mFSC values, but using associated CIs, which take into account not only the ndf, and thus the size of the mask, but also the user-decided significance level of the outcome. For generous masks the results agree very well with the traditional FSC. For very tight masks, including masks that coincide with the threshold at which the structure is visualized, the mFSC yields sensible results, while the FSC is dominated by artifacts and cannot be used. We also demonstrated that the mFSC is an ideal tool for resolution estimation of helical assemblies; that is, for systems that so far have lacked reliable quality measures. These properties of the mFSC make it easy to extend it to the estimation of local resolution. In this case, the estimation of the ndf is particularly valuable as the CI-based resolution prevents users from accepting statistically unreliable or exaggerated results, which is normally the case when the ndf is very small.

The results presented, while convincing, are only valid under the broad statistical assumptions under which mFSC calculations can be justified. The mFSC, being essentially a correlation coefficient, requires the data to be normally distributed and, in order to use Fisher’s *z*-transformation, to be based on at least 20–30 samples. In our applications the latter is almost always the case, not to mention that smaller sets would mean a very small ndf and, as a result, the CI would always yield very low resolution, so that the results would not be informative.

We have put forward arguments that normality assumptions usually hold well for cryo-EM maps. While this may be disputed for 2D projection images owing to the low electron count, 3D voxels are averages of hundreds if not thousands of samples, so that the number of added elements is more than sufficient to justify the normality assumption. Another concern is noise additivity. Owing to alignment, one would expect that higher-density regions would have lower errors (noise) than low-density regions, so the noise is correlated with the signal (3D structure). However, following our approach to noise estimation (9), we tested this possibility by correlating the difference between 80S ribosome half-maps with their sum and the result was zero. While this is reassuring, this subject requires broader studies.

Of greater concern is the independence of the Fourier coefficients. Owing to the high quality of the results, many if not most cryo-EM structures in the last decade have been determined using the maximum-likelihood (ML) method­ology (Sigworth *et al.*, 2010 ▸). Briefly, for a 3D refinement and reconstruction this means that each 2D projection image is backprojected into the 3D volume using a number of (‘probability’-weighted) in-plane translations and 3D projection directions. The number of these orientations depends mainly on the resolution of the current structure approximation. Ideally, upon convergence there would be one unique orientation per projection image, but in current implementations this is not the case. On the contrary, the number of assigned orientations appears to increase upon convergence. While on the one hand this gives the surface representation of the structure a visually appealing, smooth appearance, it also means that the Fourier components of this map are strongly correlated, even if these correlations are short-range. Worse, the natural basis for these correlations are polar coordinates (owing to the rotation of 2D projections about the origin of the system), while the resolution is computed using Fourier expansion in Cartesian coordinates. It was observed that as a result the resolution of a map computed using full ML-derived orientations of projections is higher by several Fourier pixels than the resolution of a map computed using only a proper, unique set of 2D projections of highest probability with one direction per projection (Cheng *et al.*, 2015 ▸). While this effect is easy to demonstrate, quantifications of the resolution bias of ML results are lacking. One possibility would be to enhance the mFSC methodology by additional tests of independence, for example using concepts of local correlations of 3D Fourier coefficients of maps (Sousa & Grigorieff, 2007 ▸).

In conclusion, the mFSC yields reliable results that are free of mask-induced artifacts and, owing to the inclusion of the ndf and thus reporting the resolution based on its statistical significance, the mFSC methodology reduces some of the arbitrariness associated with cryo-EM resolution estimates. This is not to say that the results are entirely objective. As we have described above, unresolved issues remain and it is still possible to bias the 3D refinement towards higher resolution values.

## Supplementary Material

Supplementary Figures. DOI: 10.1107/S2052252520011574/eh5010sup1.pdf

## Footnotes

1 In the absence of common signal, the expected value of a correlation coefficient is zero and its variance is 1/ndf. Thus, at very high frequencies where there is no common signal, the FSC values computed for quasi-independent half-volumes should have positive or negative values (with their average approaching zero), creating a visual effect of the FSC curve ‘oscillating’ about the zero axis. If the FSC values remain positive (or in some cases only negative) at high frequencies, this indicates that severe mistakes have been made in the structure-determination process (or in the manner in which the FSC is computed) and such a resolution curve cannot be used for resolution determination.
