# Supplementary material for: Reliable cryo-EM resolution estimation with modified Fourier shell correlation
Source: IUCrJ. 2020 Sep 15;7(Pt 6):995–1008. doi: 10.1107/S2052252520011574 (PMC7642792; doi:10.1107/S2052252520011574)
Supplement: Supplementary file 1 [file m-07-00995-sup1.pdf]

IUCrJ

Volume 7 (2020)

Supporting information for article:

Reliable cryo-EM resolution estimation with modified Fourier shell correlation

Pawel A. Penczek

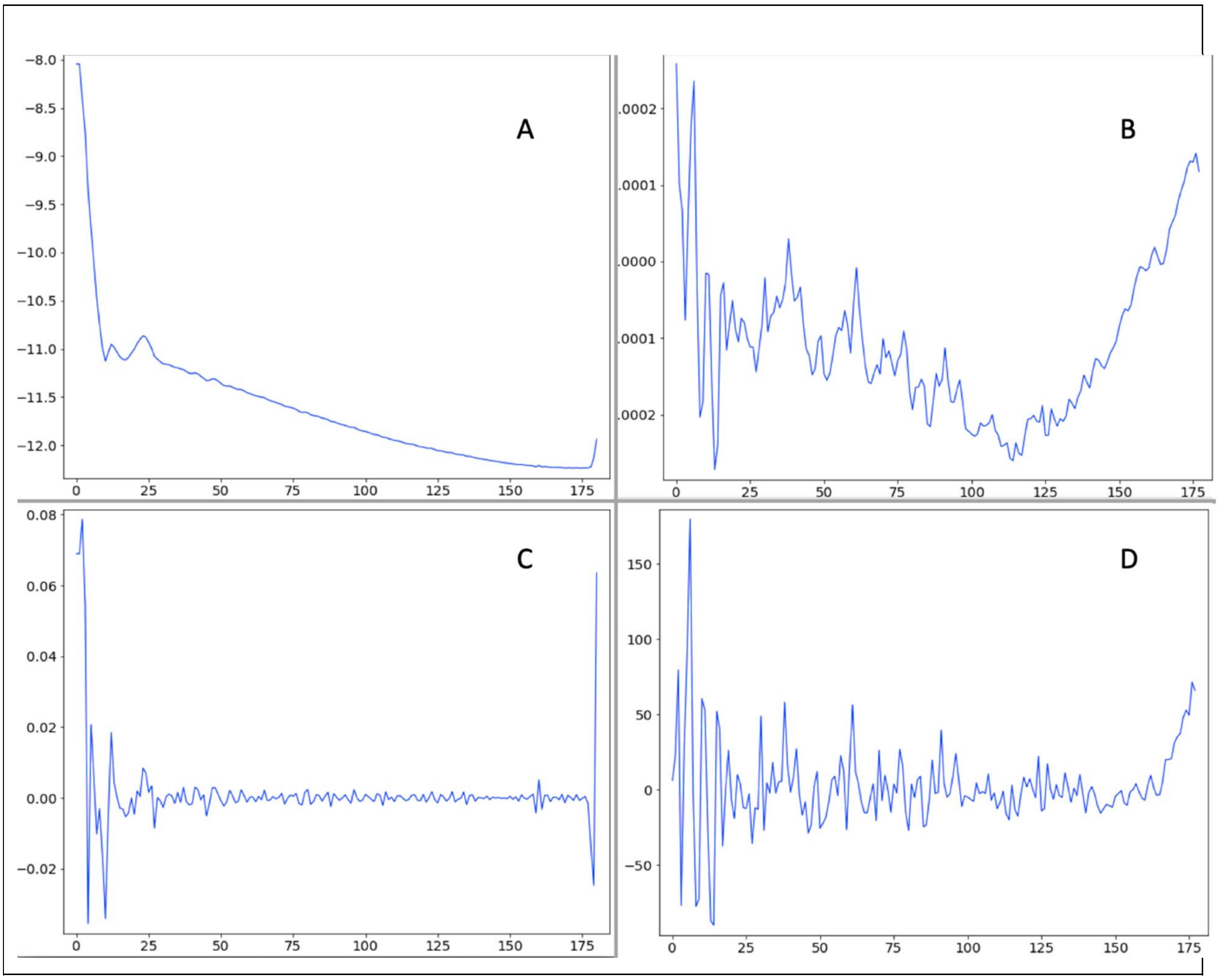

Figure S1 Prewhitening of noise in 3D map of 80S ribosome. Estimate of noise was obtained by subtracting half-maps from each other $e = u - v$ (Eq.9). (A) Logarithm of rotationally averaged power spectrum of e. (B) Rotational average of voxels values of e. (C) Logarithm of rotationally averaged power spectrum of prewhitened e. Artifacts at the beginning and end of the plot are due to: (left) small number of samples close to the origin and (right) edge effects. (D) Rotational average of voxels values after prewhitening. X-axis – distance in pixels from the volume center. Y-axis – intensities in arbitrary units.

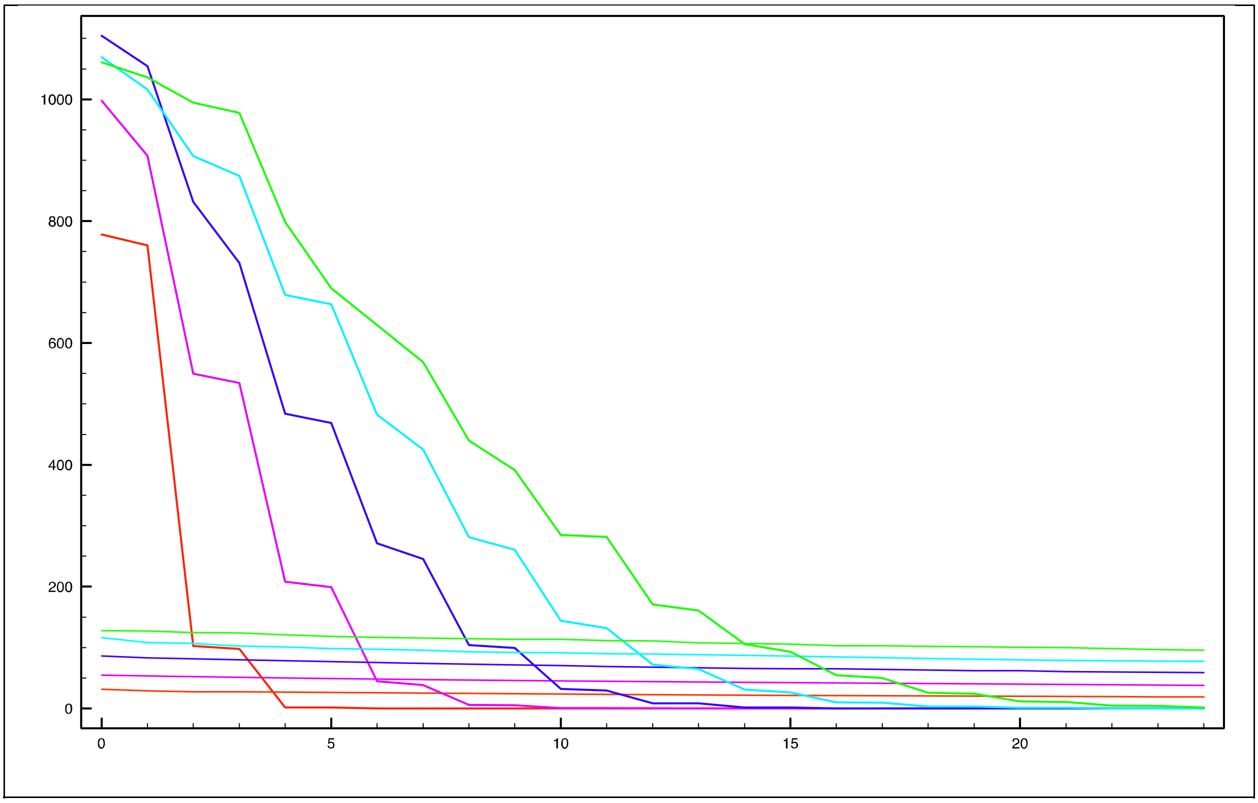

Figure S2 Determination of effective number of degrees of freedom of a random series of 128 samples multiplied by a Gaussian window function using Monte Carlo simulations. X-axis – eigenvalue number. Y-axis – eigenvalue amplitude. Falling lines – eigenvalues of Gaussian-multiplied noise realizations. Horizontal lines – eingevalues of shuffled data. σ_g is set to: red 1, magenta 2, dark blue 3, light blue 4, green 5 pixels.
